# Interaction of *HSD11B1* and *H6PD* polymorphisms in subjects with type 2 diabetes are protective factors against obesity: a cross-sectional study

**DOI:** 10.1186/s13098-019-0474-2

**Published:** 2019-09-23

**Authors:** Marcio F. Chedid, Filipe V. do Nascimento, Fernanda S. de Oliveira, Bianca M. de Souza, Cleber R. P. Kruel, Richard R. Gurski, Luis H. Canani, Daisy Crispim, Fernando Gerchman

**Affiliations:** 1 Postgraduate Program of Surgical Sciences, Universidade Federal do Rio Grande do Sul, Porto Alegre, Brazil; 2 Division of Gastrointestinal Surgery, Hospital de Clínicas de Porto Alegre, Universidade Federal do Rio Grande do Sul, Porto Alegre, Brazil; 3 Postgraduate Program in Medical Science: Endocrinology, Universidade Federal do Rio Grande do Sul (UFRGS), Porto Alegre, Brazil; 4 Division of Endocrinology and Metabolism, Hospital de Clínicas de Porto Alegre, Universidade Federal do Rio Grande do Sul (UFRGS), Rua Ramiro Barcelos 2350, Prédio 12, 4° andar, Bairro Santana, Porto Alegre, RS 90035-003 Brazil

**Keywords:** Gene expression, Cortisol, 11-beta hydroxysteroid dehydrogenase type 1, Hexose-6-phosphate dehydrogenase, Visceral, Adipose tissue

## Abstract

**Background:**

The enzyme 11-beta hydroxysteroid dehydrogenase type 1 (HSD11B1) converts inactive cortisone to active cortisol in a process mediated by the enzyme hexose-6-phosphate dehydrogenase (H6PD). The generation of cortisol from this reaction may increase intra-abdominal cortisol levels and contribute to the physiopathogenesis of obesity and metabolic syndrome (MetS). The relationship of *HSD11B1* rs45487298 and *H6PD* rs6688832 polymorphisms with obesity and MetS was studied. We also studied how *HSD11B1* abdominal subcutaneous (SAT) and visceral adipose tissue (VAT) gene expression is related to body fat distribution.

**Methods:**

Rates of obesity and MetS features were cross-sectionally analyzed according to these polymorphisms in 1006 Brazilian white patients with type 2 diabetes (T2DM). Additionally, *HSD11B1* expression was analyzed in VAT and SAT in a different cohort of 28 participants with and without obesity who underwent elective abdominal operations.

**Results:**

Although polymorphisms of the two genes were not individually associated with MetS features, a synergistic effect was observed between both. Carriers of at least three minor alleles exhibited lower BMI compared to those with two or fewer minor alleles adjusting for gender and age (27.4 ± 4.9 vs. 29.3 ± 5.3 kg/m^2^; P = 0.005; mean ± SD). Obesity frequency was also lower in the first group (24.4% vs. 41.6%, OR = 0.43, 95% CI 0.21–0.87; P = 0.019). In the second cohort of 28 subjects, *HSD11B1* gene expression in VAT was inversely correlated with BMI (r = − 0.435, P = 0.034), waist circumference (r = − 0.584, P = 0.003) and waist-to-height ratio (r = − 0.526, P = 0.010).

**Conclusions:**

These polymorphisms might interact in the protection against obesity in T2DM individuals. Obese individuals may have decreased intra-abdominal VAT *HSD11B1* gene expression resulting in decreasing intra-abdominal cortisol levels as a compensatory mechanism against central and general adiposity.

## Background

Obesity is a major public health problem causing a significant reduction in quality of life and life expectancy [1–3]. Obesity is one of the main components of the metabolic syndrome (MetS), which is characterized also by the co-occurrence of other well defined cardiovascular risk factors, including type 2 diabetes mellitus, atherogenic dyslipidemia and hypertension [4]. Weight gain promotes body fat accumulation, resulting in the activation of the inflammatory pathways, abnormal fat metabolism and the development of insulin resistance [5, 6]. The mechanisms behind this process are not clear, but in view of the similarities between individuals with Cushing’s syndrome and those with metabolic syndrome (MetS), it has been proposed that, despite normal levels of circulating cortisol, these phenotypes might arise as a consequence of tissue-specific cortisol excess due to increased activity of the enzyme 11-beta hydroxysteroid dehydrogenase type 1 (HSD11B1) [7].

Although HSD11B1 is a bidirectional enzyme in vitro, under normal circumstances it works as a reductase in the liver and adipose tissue, catalyzing the conversion of hormonally inactive cortisone into its active form, cortisol [8]. The activity of this enzyme is dependent on the provision of NADPH by the co-localized enzyme hexose-6-phosphate dehydrogenase (H6PD). In the absence of H6PD, HSD11B1 acts as a dehydrogenase, inactivating cortisol [9].

Patients with type 2 diabetes (T2DM) frequently present with obesity and visceral adiposity. Both diseases are considered among the most important determinants of a significant decrease of quality of life, cardiovascular disease and death. Transgenic mice overexpressing *HSD11B1* develop visceral obesity and exhibited insulin-resistant diabetes and dyslipidemia [10], while *HSD11B1*-knockout mice are protected from the adverse metabolic complications of obesity and high fat diet-induced hyperglycemia [11]. Moreover, HSD11B1 inhibitors improve insulin sensitivity in mice [12]. We recently systematically reviewed the relationship among *HSD11B1* abdominal adipose gene expression with obesity, T2DM and MetS in humans and reported that abdominal adipose HSD11B1 expression increased with increasing body mass index and abnormalities of glucose metabolism in most studies, but not in all, and varied with the presence of MetS. Variants of *HSD11B1* were associated with the MetS and T2DM only in specific populations [13].

Because H6PD activity determines the directionality of HSD11B1 activity, we hypothesize that polymorphisms in the *H6PD* gene might influence the effects of *HSD11B1* polymorphisms resulting in changes in gene expression related to adipose tissue metabolism, body fat distribution and the development of the MetS. Indeed, Draper et al. [14] reported that a combination of polymorphisms in the *HSD11B1* (rs45487298:delA>insA) and *H6PD* (rs6688832:G>A) genes interacts to cause cortisone reductase deficiency. They proposed a digenic triallelic mode of inheritance in which at least three minor alleles from two *loci* are necessary for trait manifestation [14].

Therefore, in the present study we investigated the potential synergistic effect of *HSD11B1* (rs45487298:delA>insA) and *H6PD* (rs6688832:G>A) polymorphisms on obesity and MetS-related characteristics in white T2DM individuals. We also investigated the relationship of HSD11B1 gene expression in abdominal subcutaneous (SAT) and visceral (VAT) adipose tissue with body fat distribution in individuals with and without MetS.

## Methods

### Participants

We performed a cross-sectional analysis of T2DM individuals who were participants of a multicenter study in Southern Brazil. This study started in 2002 with the aim of studying risk factors for T2DM and its complications. Ethnicity was mostly of European ancestry. A detailed description of this study can be found elsewhere [15]. T2DM was defined according to cut offs adopted by the American Diabetes Association (ADA).

The *HSD11B1* (rs45487298:delA>insA) and *H6PD* (rs6688832:G>A) polymorphisms were analyzed in blood samples from 1006 white T2DM individuals and compared individually for the presence of MetS. Next, MetS rates were compared according to the presence of at least three minor alleles vs. two or fewer minor alleles of these polymorphic variants. *HSD11B1* abdominal subcutaneous and visceral adipose tissue (SAT and VAT) gene expression was performed in other 28 participants with and without MetS and different degrees of glucose tolerance who underwent elective abdominal surgery. Paired samples of visceral and subcutaneous abdominal adipose tissue were collected during the procedure, cleaned with sodium chloride 0.9%, immediately frozen in liquid nitrogen and stored at − 80 °C.

The study protocol was approved by the ethics committees of the participating centers, and all individuals provided written informed consent. This study has been performed in accordance with the ethical standards as laid down in the 1964 Declaration of Helsinki.

### Clinical and anthropometric profiles and laboratory analyses

For both, the polymorphism and gene expression studies, a standard questionnaire was used to collect medical history, physical and laboratory evaluation to phenotype the study sample. For the gene expression study participants were additionally submitted to a 2-h 75-g oral glucose tolerance test with determination of plasma glucose every 30 min.

Participants were classified as having MetS using the new International Diabetes Federation criteria [16].

Serum samples were collected for laboratory testing after a 12-h fast. Glucose levels were determined using the glucose hexokinase method (Advia 1800 analyzer, Siemens Healthcare, Munich, Germany); glycated hemoglobin (A1c), by an ion-exchange HPLC procedure (Merck-Hitachi L-9100 analyzer, Darmstadt, Germany; reference range: 28–42 mmol/mol (4.7–6.0%); and total plasma cholesterol, HDL, and triglycerides, by enzymatic colorimetric methods (Advia 1800 analyzer, Siemens Healthcare, Munich, Germany). Low-density lipoprotein cholesterol (LDL) cholesterol was calculated using the Friedewald equation.

### Genetic analyses

#### Polymorphism analyses

A total of 1006 individuals were recruited for this study. From this, 998 were genotyped for the *HSD11B1* rs4548729883:delA>insA and 924 for the *H6PD* rs6688832:G>A. Genotyping success rate was 99.2% for *HSD11B1* rs4548729883:delA>insA and 91.8% for *H6PD* rs6688832:G>A. The calculated allele frequency error rate based on PCR duplicates was 0%. The consensus rate was 91.1%. Amplification reactions were performed twice.

DNA was extracted from peripheral blood leukocytes and polymorphisms were determined using primers and probes contained in the Human Custom TaqMan Genotyping Assay 40× (Assays-By-Design Service, Life Technologies, Foster City, CA, USA) as following:

*HSD11B1* rs4548729883:delA>insA: *HSD11B1*-5′-CTTACCTCCTCCTCTGAACTTTGC-3′ (forward primer), *HSD11B1*-5′-TCCTCCTGCAAGAGATGGCTATATT-3′ (reverse primer), *HSD11B1*-FAM-5′-CACCAAGAGCTTTT-3′, *HSD11B1*-VIC-5′-CACCAAAGAGCTTTT-3′.

*H6PD* rs6688832:G>A polymorphism: *H6PD*-5′-TCTGTCCGATTACTACGCCTACA-3′ (forward primer), *H6PD*-5′-GGCCATGGAAGATATGGGATAAGAG-3′ (reverse primer), *H6PD*-FAM-5′-CTGTGCGGGAGCG-3′, *H6PD*-VIC-5′-CCTGTGCAGGAGCG-3′.

Reactions were conducted in 96-well plates, in a total reaction volume of 5 µL, using 2 ng of genomic DNA, TaqMan Genotyping Master Mix 1× (Life Technologies, Foster City, CA, USA), and Custom TaqMan Genotyping Assay 1x. Plates were then placed in a real-time PCR thermal cycler (7500 Fast Real PCR System; Life Technologies, Foster City, CA, USA) and heated for 10 min at 95 °C, followed by 50 cycles of 95 °C for 15 s and 62 °C for 1 min. Fluorescence data files from each plate were analyzed using automated allele-calling software (SDS 2.1; Life Technologies, Foster City, CA, USA).

### Gene expression study

RNA was extracted from adipose tissue using Trizol^®^ reagent (Invitrogen; Life Technologies, Foster City, CA, USA). Concentration and quality of the product obtained was tested using the NanoDrop 2000 Spectrophotometer (Thermo Scientific Inc., Newark, USA). Only samples with suitable purity ratios (A260/A280 = 1.9–2.1) were used in subsequent analyses.

Real-Time reverse transcription PCR (RT-PCR) was performed in two separate steps: first, RNA was reverse transcribed into cDNA. Second, cDNA was amplified by quantitative RT-PCR (RT-qPCR). In this procedure, 1 μg of RNA was reverse transcribed to cDNA using the SuperScript^®^ III First-Strand Synthesis System for RT-PCR (Invitrogen; Life Technologies, Foster City, CA, USA) following the manufacturer’s protocol for the oligo (dT)_12–18_ method. RT-qPCR experiments were performed in a 7500 Fast Real-Time PCR System Thermal Cycler with 7500 FAST Sequence Detection System Software (Life Technologies, Foster City, CA, USA) monitoring the increase in fluorescence of the SYBR^®^ green dye [17]. Each sample was analyzed in triplicate and a negative control was added in each experiment.

Target and reference gene amplifications showed similar efficiencies in all experiments (E = 95% to 105%) allowing the use of this method. Primers for *HSD11B1* (target gene) and *β*-*actin* (reference gene) were designed using the Primer Express 3.0 software (Life Technologies): *HSD11B1*: 5′-GCTGCCTTGCCCATGCT-3′ (forward primer), 5′-CAGCCAGAGAGGAGACGACAA-3′ (reverse primer); *β*-*actin*: 5′-GCGCGGCTACAGCTTCA-3′ (forward primer), 5′-CTTAATGTCACGCACGATTTCC-3′ (reverse primer).

RT-qPCR reactions were performed using 10 μL 2× Fast SYBR^®^ Green Master Mix (Life Technologies, Foster City, CA, USA), 1 μL (1 ng/μL) of primers for *HSD11B1* or *β*-*actin*, 1 μL (1 μg/μL) of cDNA, in a total volume of 20 μL. The reaction conditions were: initial cycle of 20 s at 95 °C, followed by 50 cycles at 95 °C for 3 s and 60 °C for 1 min. RT-qPCR specificity was determined using melting curve analyses and all primers generated amplicons that produced a single sharp peak during the analyses.

The measurement of *HSD11B1* expression was performed by relative quantification using the comparative ΔΔCq method and expressed relative to the reference gene (*β*-*actin*) [18]. Validation tests were carried out by amplification of the target gene (*HSD11B1*) and the reference gene (*β*-*actin*), using serial dilutions of cDNA samples. The ΔΔCq method calculates changes in gene expression as relative fold changes (n-fold change) between the experimental sample and an external calibrator.

### Statistical analyses

Results are expressed as means and standard deviations (SD), percentages, or median (interquartile range). Clinical and laboratory characteristics were compared using analysis of variance (ANOVA), the unpaired Student’s *t* test, Pearson’s correlation or χ^2^ as appropriate. Variables with skewed distribution were log-transformed. Bonferroni corrections were used to account for multiple comparisons. Departures from the Hardy–Weinberg equilibrium were verified using the Chi-square test (χ^2^). The relationship of *HSD11B1* rs45487298:delA>insA and *H6PD* rs6688832:G>A polymorphisms with different MetS-related components were tested by general linear model univariate analyses, adjusting for covariates.

Using the digenic triallelic mode of inheritance proposed by Draper et al. we analyzed the combined effect of at least three minor alleles of the *HSD11B1* rs45487298:delA>insA and *H6PD* rs6688832:G>A polymorphisms in modulating different MetS-related characteristics: systolic and diastolic BP, BMI, WC, HDL, and triglyceride levels [14]. Multiple logistic regression analysis was performed with the presence of obesity as the dependent variable and age, gender, and presence of at least three minor alleles of the two analyzed polymorphisms as independent variables.

It was calculated a sample size (WinPepi program, version 11.39, London, UK) of 15 subjects to compare the relationship between SAT and VAT *HSD11B1* expression using a significance level of 5%, power of 90%, 1 to 1 proportion between insulin sensitive and resistant individuals based on the follow study [19].

All analyses were performed in the SPSS 18.0 (SPSS Inc., Chicago, USA). P < 0.05 was considered significant.

## Results

### Relationship between polymorphisms and MetS-related parameters

The 1006 T2DM included in the study were 58.4 ± 10.2 years old, 43.0% males, known T2DM duration of 11.6 ± 8.6 years, BMI 29.1 ± 5.2 kg/m^2^ (obesity = 40.3%), 63.6% had hypertension, and 76.3% had MetS. In the gene expression study 16.6% of subjects were lean, 25% were overweight and 58.4% were obese.

Of the 998 individuals genotyped for the *HSD11B1* rs45487298:delA>insA polymorphism, 654 (65.5%) individuals were homozygous for the delA allele (delA/delA), 311 (31.2%) were heterozygous (delA/insA), and 33 (3.3%) were homozygous for the insA allele (insA/insA). Genotypes were in agreement with those predicted by Hardy–Weinberg equilibrium (P > 0.05). The insA allele frequency was 0.189. Table 1 summarizes the clinical and laboratory data of the individuals grouped according to the *HSD11B1* rs45487298:delA>insA polymorphism. Systolic and diastolic BP, BMI, WC, total cholesterol, HDL, LDL, and triglyceride levels were not significantly different among the three genotypes. It bears mentioning that none of these variables exhibited significant differences when assuming dominant (insA/insA + insA/delA vs. delA/delA) or recessive (insA/insA vs. insA/delA + delA/delA) models of inheritance for the insA allele (data not shown).

**Table 1:** Clinical and laboratory characteristics of T2DM patients according to genotypes of the HSD11B1 rs45487298:delA>insA polymorphism

	*HSD11B1* rs45487298:delA>insA polymorphism			F/P*	P^†^
	DelA/DelA (n = 654)	DelA/InsA (n = 311)	InsA/InsA (n = 33)		
Age (years)	58.5 ± 10.6	58.2 ± 10.0	57.3 ± 8.9	–	0.642
Males (%)	42.8	43.4	54.5	–	0.415
T2DM duration (years)	10.0 (1–48)	10.0 (1–51)	12.5 (1–46)	–	0.330
Systolic BP (mmHg)^a^	137.7 ± 22.6	133.1 ± 20.2	131.0 ± 13.8	2.952/0.053	–
Diastolic BP (mmHg)^a^	84.7 ± 12.2	83.3 ± 12.8	83.0 ± 7.6	0.591/0.554	–
Body mass index (kg/m^2^)^b^	29.2 ± 5.0	29.3 ± 5.6	28.4 ± 4.9	0.250/0.778	–
Waist circumference (cm)^b^	98.7 ± 12.3	98.7 ± 11.6	100.8 ± 14.4	0.141/0.868	–
HbA1c (%)	7.2 ± 2.1	7.4 ± 2.1	7.3 ± 2.3		0.909
Total cholesterol (mg/dL)^b^	208.8 ± 46.4	208.8 ± 50.3	193.3 ± 54.1	0.682/0.384	–
HDL cholesterol (mg/dL)^c^	46.4 ± 11.6	50.3 ± 11.6	42.5 ± 19.3	2.488/0.116	–
LDL cholesterol (mg/dL)^c^	135.3 ± 50.3	131.5 ± 50.3	112.1 ± 23.2	1.889/0.155	–
Triglycerides (mg/dL)^c^	141.7 (26.6–1470.3)	150.6 (26.6–779.5)	230.3 (44.3–806.0)	0.280/0.756	–
MetS (%)	78.8	73.4	78.6	–	0.232

Data expressed as mean ± SD, median (range), or %

*BP* blood pressure, *MetS* metabolic syndrome, *T2DM* type 2 diabetes mellitus, *HbA1c* glycated hemoglobin

* F and P values obtained from the general linear model univariate analyses, after adjusting for: ^a^age and gender, use of medication for hypertension, and BMI; ^b^age and gender; ^c^age, gender and use of hypolipidemic medications

^†^P values were computed by χ^2^ test or ANOVA as appropriate. Only P values lower than the Bonferroni threshold (0.0055) were considered statistically significant

Four-hundred and sixty-four (50.2%) individuals had the *H6PD* G/G genotype, 377 (40.8%) the A/G genotype, and 83 (9.0%) the A/A genotype of the rs6688832:G>A polymorphism. All genotypes were in Hardy–Weinberg equilibrium (P > 0.05), and the A allele frequency was 0.294. Table 2 illustrates the clinical and laboratory data of the individuals grouped according to the *H6PD* rs6688832:G>A polymorphism. This polymorphism had no statistically significant relationship with the MetS-related characteristics listed in Table 2. Furthermore, none of these variables exhibited significant differences when assuming dominant (A/A + G/A vs. G/G) or recessive (A/A vs. G/A + A/A) models of inheritance for the A allele (data not shown). Clinical and laboratory characteristics of T2DM individuals grouped according to the presence of at least three minor alleles of *HSD11B1* rs45487298:delA>insA and *H6PD* rs6688832:G>A polymorphisms are shown in Table 3. Taking into consideration a Bonferroni threshold of 0.0055 (P = 0.05 divided by 9 MetS-related features analyzed in Table 3), we observed that BMI was significantly lower in carriers of at least three minor alleles of the *HSD11B1* and *H6PD* polymorphisms as compared to individuals with fewer than three minor alleles, adjusting for age and gender (P = 0.005). No significant gene–gene interaction was observed between the *HSD11B1* rs45487298:delA>insA and *H6PD* rs6688832:G>A polymorphisms in modulating other MetS-related features (Table 3). As expected, the frequency of obesity was lower among individuals carrying at least three minor alleles of *HSD11B1* rs45487298:delA>insA and *H6PD* rs6688832:G>A polymorphisms than in the group of individuals with fewer than three minor alleles (24.4% vs. 41.6%, respectively; P = 0.033). Logistic regression analysis, with adjustment for age and gender, confirmed that the presence of at least three minor alleles of both polymorphisms is an independent protection factor against obesity in patients with T2DM (OR = 0.43, 95% CI 0.21–0.87, P = 0.019).

**Table 2:** Clinical and laboratory characteristics of T2DM patients according to genotypes of the *H6PD* rs6688832:G>A polymorphism

	*H6PD* rs6688832:G>A polymorphism			F/P*	P^†^
	G/G (n = 464)	G/A (n = 377)	A/A (n = 83)		
Age (years)	59.0 ± 10.3	58.0 ± 10.6	56.7 ± 10.4	–	0.125
Males (%)	44.2	44.0	38.6	–	0.621
T2DM duration (years)	11 (1–47)	10 (1–51)	10 (1–48)	–	0.043
Systolic BP (mmHg)^a^	135.4 ± 21.3	136.9 ± 22.2	136.0 ± 24.0	1.401/0.242	–
Diastolic BP (mmHg)^a^	83.4 ± 11.2	85.0 ± 13.5	84.7 ± 13.0	2.656/0.071	–
Body mass index (kg/m^2^)^b^	29.1 ± 5.2	29.4 ± 5.4	28.6 ± 5.2	1.647/0.193	–
Waist circumference (cm)^b^	98.6 ± 12.6	98.5 ± 11.9	98.7 ± 10.7	0.003/0.997	–
HbA1c (%)	7.1 ± 2.1	7.2 ± 2.1	7.4 ± 2.3		0.196
Total cholesterol (mg/dL)^c^	208.8 ± 50.3	204.9 ± 42.5	220.4 ± 42.5	1.353/0.261	–
HDL cholesterol (mg/dL)^c^	46.4 ± 15.5	46.4 ± 11.6	46.4 ± 11.6	1.339/0.264	–
LDL cholesterol (mg/dL)^a^	131.5 ± 54.1	131.5 ± 42.5	3.7 ± 38.7	0.657/0.520	–
Triglycerides (mg/dL)^c^	150.7 (26.6–1470.3)	141.7 (0.3–1240.0)	150.7 (53.1–469.4)	0.414/0.661	–
MetS (%)	74.4	76.7	75.4	–	0.790

Data expressed as mean ± SD, median (range), or %

*BP* blood pressure, *MetS* metabolic syndrome, *T2DM* type 2 diabetes mellitus, *HbA1c* glycated hemoglobin

* F and P values obtained from the general linear model univariate analyses, after adjusting for: ^a^age and gender, use of medication for hypertension, and BMI; ^b^age and gender; ^c^age, gender, use of hypolipidemic medications

^†^P values were computed by χ^2^ test or ANOVA as appropriate. Only P values lower than the Bonferroni threshold (0.0055) were considered statistically significant

**Table 3:** Interaction analyses between *HSD11B1* rs45487298:delA>insA and *H6PD* rs6688832:G>A polymorphisms in T2DM patients

	rs45487298:delA>insA–rs6688832:G>A interaction		F/P*	P^†^
	< 3 minor alleles (n = 871)	≥ 3 minor alleles (n = 45)		
Age (years)	58.6 ± 10.5	54.9 ± 8.9	–	0.021
Males (%)	43.2	51.1	–	0.370
T2DM duration (years)	10 (1–51)	10 (1–46)	–	0.656
Systolic BP (mmHg)^a^	136.5 ± 22.1	128.8 ± 18.2	0.553/0.457	–
Diastolic BP (mmHg)^a^	84.3 ± 12.6	81.7 ± 7.9	0.048/0.826	–
Body mass index (kg/m^2^)^b^	29.3 ± 5.3	27.4 ± 4.9	7.856/0.005	–
Waist circumference (cm)^b^	98.7 ± 12.0	97.5 ± 13.5	0.526/0.469	–
HbA1c (%)	7.2 ± 2.1	7.3 ± 2.1		0.678
Total cholesterol (mg/dL)^c^	208.8 ± 46.4	204.9 ± 46.4	0.179/0.673	–
HDL cholesterol (mg/dL)^c^	46.4 ± 11.6	46.4 ± 11.6	3.112/0.079	–
LDL cholesterol (mg/dL)^c^	135.3 ± 46.4	131.5 ± 61.9	0.084/0.775	–
Triglycerides (mg/dL)^c^	150.6 (26.6–1470.4)	124 (53.1–806.0)	1.102/0.295	–
MetS (%)	75.9	68.9	–	0.398

Data expressed as mean ± SD, median (range), or %

*BP* blood pressure, *MetS* metabolic syndrome, *T2DM* type 2 diabetes mellitus, *HbA1c* glycated hemoglobin

* F and P values obtained from the general linear model univariate analyses, after adjusting for: ^a^age and gender, use of medication for hypertension, and BMI; ^b^age and gender; ^c^age, gender, use of hypolipidemic medications

^†^P values were computed by χ^2^ test or Student’s t-test as appropriate. Only P values lower than the Bonferroni threshold (0.0055) were considered statistically significant

None of the analyzed variables exhibited significant differences among individuals carrying at least two minor alleles of *HSD11B1* rs45487298:delA>insA and *H6PD* rs6688832:G>A polymorphisms and individuals with fewer than two minor alleles (data not shown). Furthermore, taking into account that only three individuals had four rs45487298:delA>insA and rs6688832:G>A minor alleles, it was not possible to evaluate if the protection to obesity could increase on those individuals carrying all minor alleles of the two genes.

### Relationship between *HSD11B1* gene expression in abdominal subcutaneous and visceral adipose tissue with anthropometric parameters

There were no difference in *HSD11B1* abdominal SAT and VAT gene expressions between obese (BMI > 30 kg/m^2^) and non-obese individuals. Also there were no differences in *HSD11B1* abdominal SAT and VAT gene expressions between patients with and without MetS (Table 4).

**Table 4:** Abdominal *HSD11B1* adipose tissue gene expression stratified by the presence of obesity or MetS

	SAT (n = 18)	VAT (n = 24)
Non obese	1.04 (0.39–6.05)	3.01 (0.39–3.74)
Obese	0.85 (0.37–9.54)	1.32 (0.63–3.47)
MetS (−)	0.9 (0.6–10.36)	2.39 (0.69–24.73)
MetS (+)	0.97 (0.3–5.65)	1.30 (0.39–3.25)

Data expressed as median (P25–P75)

*SAT* subcutaneous adipose tissue, *VAT* visceral adipose tissue, *MetS (−)* subjects without metabolic syndrome, *MetS (+)* subjects with metabolic syndrome

Gene expression did not differ among the groups (P > 0.05). P values were computed by Student’s t-test

*HSD11B1* SAT expression was not related with BMI, waist circumference, and waist-to-height ratio (Table 5). In contrast VAT expression was inversely related to all of these parameters (adjusting for age and gender). In order to better understand these relationships, we stratified the sample by body size and MetS presence. *HSD11B1* VAT but not SAT expression was inversely related to BMI, WC and WHtR in participants with BMI ≥ 30 kg/m^2^. However, both VAT and SAT expression were not related to these parameters in those with BMI < 30 kg/m^2^. When stratifying the study sample by the presence of MetS, *HSD11B1* VAT expression was inversely related to all these anthropometric parameters, being statistically significant only for WC and WHtR in the MetS+ group. Although there was a trend towards SAT expression to be related to BMI, WC and WHtR in those without the MetS, it did not reach statistical significance. For MetS participants, SAT expression was not related to these anthropometric parameters (Table 5).

**Table 5:** Correlations between *HSD11B1* gene expression in adipose tissue and anthropometric parameters in all, non-obese, obese, MetS (−) and MetS (+) subjects

	SAT					VAT				
	All (n = 18)	Non obese (n = 8)	Obese (n = 10)	MetS (−) (n = 6)	MetS (+) (n = 12)	All (n = 24)	Non obese (n = 11)	Obese (n = 13)	MetS (−) (n = 8)	MetS (+) (n = 16)
BMI	0.077 (0.763)	− 0.454 (0.258)	0.482 (0.158)	0.790 (0.061)	− 0.332 (0.291)	− 0.435 (0.034)^a^	− 0.489 (0.127)	− 0.585 (0.036)^a^	− 0.483 (0.225)	− 0.411 (0.114)
WC	0.178 (0.494)	0.100 (0.813)	0.317 (0.406)	0.744 (0.090)	− 0.308 (0.356)	− 0.584 (0.003)^b^	− 0.478 (0.137)	− 0.728 (0.007)^b^	− 0.546 (0.161)	− 0.597 (0.019)^a^
WHtR	− 0.014 (0.956)	− 0.189 (0.653)	0.140 (0.719)	0.797 (0.057)	− 0.448 (0.167)	− 0.526 (0.010)^b^	− 0.122 (0.721)	− 0.773 (0.003)^b^	− 0.525 (0.181)	− 0.539 (0.038)^a^

Data expressed as Pearson’s correlation (P value)

*SAT* subcutaneous adipose tissue, *VAT* visceral adipose tissue, *BMI* body mass index, *WC* waist circumference, *WHtR* waist-to-height ratio, *MetS (−)* subjects without metabolic syndrome, *MetS (+)* subjects with metabolic syndrome

^a^P < 0.05; ^b^P ≤ 0.01

## Discussion

This study investigated the association of the *HSD11B1* rs45487298:delA>insA and *H6PD* rs6688832:G>A polymorphisms with MetS-related characteristics in white subjects with T2DM. When independently analyzed, neither of these two polymorphisms was associated with any feature of MetS. However, when analyzing the rs45487298:delA>insA and rs6688832:G>A polymorphisms in combination, we observed a significant gene–gene interaction modulating the risk of obesity in T2DM individuals carrying at least three minor alleles of the two polymorphisms.

There is compelling biochemical evidence of cooperativity between H6PD and HSD11B1 enzymes [9, 20]. Lavery et al. [21] showed that *H6PD* knockout mice have a profound switch in HSD11B1 activity from oxoreductase to dehydrogenase, increasing corticosterone clearance and resulting in a reduction in circulating corticosterone levels. This demonstrates a critical requirement of H6PD for HSD11B1 oxoreductase activity.

The effect of this interaction has been previously associated with risk of cortisone reductase deficiency [14]. As suggested by Draper et al. [14], this interaction might occur because both rs45487298:delA>insA and rs6688832:G>A polymorphisms seem to have functional effects. The rs45487298:delA>insA polymorphism is located in an enhancer region of intron 3 of the *HSD11B1* gene. It has been associated with decreased *HSD11B1* expression in vivo and after transfection of minigene constructs in cultured cells [14]. Draper et al. [14] also reported that in cell cultures the rs6688832:G>A polymorphism in the *H6PD* gene decreased the enzyme activity to less than 50% of normal, impairing the generation of reduced NADPH and, consequently, reducing HSD11B1 activity. Nevertheless, Lavery et al. have not found a reduction in H6PD enzyme activity by the rs6688832:G>A variant [22].

Previous studies have suggested an association between different *HSD11B1* gene polymorphisms and MetS features [23–27]. The rs45487298:delA>insA polymorphism was associated with higher BMI, altered body composition and insulin resistance in obese US children [25]. Nair et al. [26] reported an association between the rs846910:G>A and rs12086634:A>G polymorphisms in the *HSD11B1* gene and risk of T2DM in Pima Indians. Devang et al. also revealed that the HSD11B1 rs846910 AG contributed to an increased risk of T2D in South Indians. The authors also indicated that HSD11B1 rs12086634 TG contributed to an increased risk of both T2D and MetS [27]. Gandhi et al. detected a significant association between the HSD11B1 gene polymorphism (rs12086634) and occurrence of MetS compared to controls [28].

In contrast, other studies showed no association between *HSD11B1* gene polymorphisms and body composition, glucose metabolism or MetS [13]. White et al. [29] did not find any association between *HSD11B1* rs12086634:T>G and *H6PD* rs6688832:G>A polymorphisms (either separately or in combination) and BMI, waist-to-hip ratio, visceral adiposity, measures of insulin sensitivity or risk of polycystic ovary syndrome (PCOS) in a population-based sample from the Dallas Heart Study. Furthermore, Draper et al. [30] showed no association of the *HSD11B1* rs12086634 variant (which is in complete linkage disequilibrium with the rs45487298:delA>insA variant) and *H6PD* rs6688832 variant with susceptibility to BMI and waist-to-hip ratio in a UK study with 213 women with PCOS and 549 controls.

Recently, Moon et al. reported that *HSD11B1* rs12086634:A>G and rs1000283:C>T polymorphisms were associated with MetS in T2DM individuals, while the *H6PD* rs17368528:C>T polymorphism was a risk factor for MetS in non-diabetic South Koreans [31]. However, differently from what we found in our study, no significant association of these SNPs with type 2 diabetes and metabolic syndrome was found after considering the multiple comparisons in the total study population. Therefore, HSD11B1 and H6PD polymorphisms analyzed individually may not be associated with type 2 diabetes and MetS.

The inconsistent results reported by the studies cited herein may be at least partly explained by differences in study designs, sample sizes, ethnicity, and analyzed HSD11B1 polymorphisms. Moreover, a number of studies analyzed only the expression of polymorphisms of the HSD11B1 gene. Thus, such studies may not have observed associations of the interaction of HSD11B1 gene polymorphisms to certain H6PD polymorphisms with MetS-related characteristics. There might also be additional polymorphisms in these two genes that were not identified in previous studies, but could have major effects on HSD11B1 gene expression or enzyme activity.

Despite inconsistent results regarding associations between *HSD11B1* gene polymorphisms and MetS-related characteristics, a compelling evidence base argues for HSD11B1 as a major etiological factor in obesity and related features [9, 32, 33]. In addition, modulation of HSD11B1 activity has also an effect on multiple target tissues which promote insulin resistance independently of obesity. For example, in lean glucose-intolerant individuals, adipose HSD11B1 activity is not increased and hepatic HSD11B1 activity is maintained [34] compared with the downregulation of hepatic HSD11B1 that occurs in obesity [35]. Inhibition of HSD11B1 with oral carbenoxolone enhances hepatic insulin sensitivity [36], and has a greater effect in non-obese glucose-intolerant participants than in healthy people [37]. Moreover, the expression of HSD11B1 in myoblast cultures stimulated with glucocorticoids was negatively correlated with insulin sensitivity [38]. Consequently, HSD11B1 is a promising target for pharmacological inhibition in individuals with T2DM and/or MetS [9].

In this context, overexpression of the *HSD11B1* gene might determine an increase in cortisol generation and, secondarily, generate pro-obesity effects. Therefore, we hypothesize that the interaction between *HSD11B1* rs45487298:delA>insA and *H6PD* rs6688832:G>A polymorphisms may generate a significant decrease in HSD11B1 levels in adipocytes and other tissues; and consequently, this may decrease the risk of obesity in individuals with T2DM who carry at least three minor alleles of these polymorphisms. However, taking into account the controversial functional studies regarding the *H6PD* rs6688832:G>A effect on H6PD activity [14, 22], it seems possible that this polymorphism might be only a neutral polymorphic variant in linkage disequilibrium with an unknown causative mutation to be found elsewhere in the *H6PD* gene. In addition, in view that has been previously shown differences in regulation of glucocorticoid activity in individuals with and without impaired glucose tolerance [39–41], the present data should be interpreted with caution when translated to non-glucose-tolerant obese participants.

Data of *HSD11B1* gene expression and its association with anthropometric parameters are conflicting due to the large difference between the studied populations. Studies performed in participants without MetS have shown that *HSD11B1* abdominal adipose tissue expression is positively related with BMI and measurements of central adiposity such as waist circumference [42, 43] whereas *HSD11B1* gene expression was not related or inversed related to anthropometric parameters in participants with MetS [38, 44].

While we found no relationships of *HSD11B1* gene expression in abdominal SAT with measurements of adiposity distribution, namely BMI, WC and WHtR, the expression of this gene in abdominal VAT was inversely and significantly related to these parameters. When stratifying the sample either by the presence of obesity or MetS, in both groups we have found an inverse relationship between abdominal adipose tissue *HSD11B1* gene expression and BMI, but this relationship remained significant only in those with obesity. Lutz et al. investigated the expression of nine different polymorphisms for the HSD11B1 gene in a total of 327 German patients [45]. The major C allele of rs2235543 and the major G allele of rs12565406 were significantly associated with increased VAT mass. It would be interesting to have analyzed these polymorphic variants in participants of our sample in which adipose tissue gene expression was studied. However, due to the low frequency of the minor allele of *HSD11B1* in the polymorphism study sample (0.33% of homozygozity), it would be unlikely to have enough number of subjects identified as having this allelic combination in the 28 participants which were studied in our gene expression cohort. As a result, even with a common direction of the polymorphism and gene expression studies, we believed that would be interesting to have a future studied in which polymorphism and gene expression analysis will be performed in the same subjects of a larger sample size population, what we, unfortunately, were not able to do it.

The results of the present study suggest that *HSD11B1* abdominal adipose tissue expression in participants with obesity and/or MetS decreases in order to compensate increased HSD11B1 activity. Although we did not tested this hypothesis, we believe this may explain in part why T2DM participants carrying at least three minor alleles of *HSD11B1* rs45487298:delA>insA and *H6PD* rs6688832:G>A exhibit lower BMI than those carrying fewer than three minor alleles.

## Conclusions

We have shown that the *HSD11B1* rs45487298:delA>insA and *H6PD* rs6688832:G>A polymorphisms might interact in protecting against obesity in T2DM individuals. Further research is required to provide functional analyses of the effects of these polymorphisms on the pathogenesis of obesity and MetS, and to confirm this result in other populations. Since HSD11B1 inhibition is now recognized as a promising pathway for pharmacological treatment of obesity, T2DM and MetS, our results might have an importance in future pharmacogenetics studies regarding the clinical testing of genetic variations that could give rise to different responses to HSD11B1 inhibitors.

## Data Availability

The datasets used and/or analyzed during the current study are available from the corresponding author on reasonable request.

## Abbreviations

ANOVA: analysis of variance
A1c: glycated hemoglobin
HPLC: high-performance liquid chromatography
HSD11B1: 11-beta hydrosteroid dehydrogenase type 1
H6PD: glucose-6-phosphate dehydrogenase
LDL: low-density lipoprotein cholesterol cholesterol
MetS: metabolic syndrome
NADPH: nicotinamide adenine dinucleotide phosphate
RT-qPCR: quantitative RT-PCR
SAT: subcutaneous abdominal tissue
SD: standard deviations
T2DM: type 2 diabetes
VAT: visceral abdominal tissue
